# Equity in Full Immunization Coverage Among Children Aged 12-23 Months in Urban and Rural Areas of Hyderabad, India: A Community-Based Cross-Sectional Study

**DOI:** 10.7759/cureus.117546

**Published:** 2026-10-06

**Authors:** Shadan Butool, Rafath Unnisa Begum, Prafful K Butti, Tameema Kauser, Hafsa Sayeed, Sreeha Baddam

**Affiliations:** 1 Department of Community Medicine, Gandhi Medical College and Hospital, Secunderabad, IND; 2 Department of Community Medicine, Government Medical College, Sangareddy, IND; 3 Department of Community Medicine, Government Medical College, Siddipet, IND; 4 Department of Ophthalmology, Osmania Medical College, Hyderabad, IND; 5 Department of Pathology, Osmania Medical College, Hyderabad, IND; 6 Department of General Surgery, Nizamabad Medical College, Nizamabad, IND

**Keywords:** child immunization, health inequality, sociodemographic factors, socioeconomic disparities, vaccination coverage

## Abstract

Background: Persistently low and inequitable childhood immunization coverage remains an important public health concern, particularly among socially and economically disadvantaged populations. Field-practice-level data that align with the national surveys are needed to understand local drivers of immunization inequity.

Objectives: This study aimed to determine the association between selected sociodemographic factors and full immunization status, to assess the extent and pattern of socioeconomic inequality in full immunization coverage, and to describe disparities by gender, geographic location, and urban-rural residence among children aged 12-23 months.

Methods: A community-based cross-sectional study was conducted from August to October 2023 among 400 children aged 12-23 months (200 urban, 200 rural), selected by multistage random sampling from the field-practice areas of Osmania Medical College, Hyderabad, Telangana, India. Full immunization status was verified against Mother and Child Protection Cards and Anganwadi Centre records. Associations were assessed using the chi-squared test; socioeconomic inequality was quantified using the Erreygers Concentration Index, and Fairlie decomposition was used to identify contributors to the socioeconomic gap.

Results: Among 400 children, 228 (57.0%) were fully immunized and 172 (43.0%) were non-fully immunized. Full immunization was significantly associated with socioeconomic status (SES), place of residence, sex, and birth order. Coverage was higher among children from the upper socioeconomic class (152/224, 67.86%) than the lower socioeconomic class (76/176, 43.18%; p<0.001), among urban (130/200, 65.0%) than rural children (98/200, 49.0%; p=0.002), and among males (140/226, 61.95%) than females (88/174, 50.57%; p=0.030). Maternal age, maternal education, and religion were not significantly associated with immunization status. A pro-rich distribution was evident (Concentration Index +0.1067; Erreygers Index +0.2432; Wagstaff Index +0.2481), with an absolute SES gap of 24.68 percentage points. Fairlie decomposition explained 1.96 percentage points (7.9%) of the observed gap; urban residence (+136.9%) and Hindu religion (+60.1%) widened the gap, whereas first birth order (−53.1%), male sex (−25.4%), and maternal literacy (−24.8%) narrowed it. The SES equity gap was wider among females (+34.15 percentage points) than males (+18.57) and among urban (+52.74) than rural children (−5.11). Closing the SES gap could increase overall coverage by 10.86 percentage points, corresponding to a population attributable fraction of 16.0%.

Conclusion: Full immunization coverage in this population remains below global targets and shows a pattern by SES, residence, and gender, with disadvantage compounding among lower-SES girls and lower-SES urban households. Because measured sociodemographic factors explained less than one-tenth of the observed inequality, closing this gap will likely require health system and supply-side interventions alongside household-targeted approaches.

## Introduction

Immunization is among the most successful and cost-effective public health interventions in history. Incomplete immunization increases children's susceptibility to vaccine-preventable infections, which can worsen nutritional deficiencies and perpetuate a cycle of infection, malnutrition, and increased mortality risk [1]. Immunization prevents an estimated 3.5-5 million deaths annually and contributes substantially to global health, social well-being, and economic development [2]. Every dollar invested in immunization in low- and middle-income countries is estimated to generate approximately USD 16 in direct economic benefits, rising to an estimated USD 44 per dollar invested when broader societal gains such as increased life expectancy are included [3].

Coverage rose markedly following the launch of the Expanded Programme on Immunization (EPI) in 1974, but global immunization coverage stagnated, with diphtheria-tetanus-pertussis (DTP3) coverage remaining around 85% between 2010 and 2019 before declining during the COVID-19 pandemic [4]. In 2021, approximately 25 million children did not receive the third dose of the DTP3 vaccine, including 18.2 million "zero-dose" children who received no DTP-containing vaccine, highlighting persistent inequities in access to routine immunization and the need to investigate their underlying structural determinants [5,6].

The Immunization Agenda 2030 (IA2030) and Gavi's 5.0 Strategy have emphasized "leaving no one behind," with a particular focus on zero-dose and under-immunized communities [4]. Health equity requires that every individual utilize their right to quality healthcare without being disadvantaged by personal attributes such as gender, socioeconomic status (SES), or geographic location [7]. Global immunization disparities are shaped by the intersection of SES and gender, with poverty, maternal education, and limited maternal decision-making power all influencing children's access to, and completion of, vaccination [8]. Evidence from India suggests that maternal education may contribute more to socioeconomic inequality in full immunization than household income alone, highlighting the need for interventions that address health literacy and gender-related barriers alongside economic disadvantage [9]. Socioeconomic determinants that influence health-seeking behavior may shape access to and uptake of childhood immunization. These socioeconomic inequalities can be quantified using concentration-based indices, which assess how a health outcome is distributed across the socioeconomic ranking of a population. The Concentration Index (CI) summarizes the extent and direction of socioeconomic-related inequality, with positive values indicating a concentration of the health outcome among higher socioeconomic groups and negative values indicating a concentration of the health outcome among lower socioeconomic groups. However, when the health outcome is binary or bounded, such as full immunization status, the standard CI has limitations in interpretation and comparability because it depends on the mean of the outcome. The Erreygers Index (EI) provides a correction for bounded outcomes and facilitates a more appropriate assessment of socioeconomic-related inequality in binary health outcomes. Despite increasing attention to socioeconomic disparities in childhood immunization, there remains limited evidence examining both the magnitude and distribution of these inequalities using concentration-based measures in urban and rural field-practice populations in Hyderabad. Therefore, this study assessed socioeconomic inequality in full immunization coverage using the CI and the EI, along with the absolute socioeconomic gap and decomposition of the observed disparity.

Socioeconomic conditions, family income, parental education, and gender-related factors are among the principal determinants of complete and timely immunization [10]. Identifying and addressing these barriers can support policymakers in advancing equitable immunization coverage and progress towards universal health coverage (UHC). Previous studies in India have consistently shown that socioeconomic and demographic factors influence childhood immunization coverage, with children from poorer households and less-educated mothers generally experiencing lower coverage [1,2,4,11]. National-level analyses have documented socioeconomic and rural-urban inequalities in coverage and identified maternal education, household wealth, place of residence, and access to health services as important contributors to these disparities. However, national surveys may mask local and within-community inequalities, particularly differences between urban and rural populations within a defined field-practice setting, a gap this study aims to address.

Objectives

This study aimed to determine the association between selected sociodemographic factors and full immunization status among children aged 12-23 months, to assess the extent and pattern of socioeconomic inequalities in full immunization coverage among children aged 12-23 months, and to quantify the contribution of selected sociodemographic factors to the observed socioeconomic gap in full immunization coverage among children aged 12-23 months in urban and rural field-practice areas of Hyderabad.

## Materials and methods

Study design and setting

A community-based cross-sectional study was conducted from August to October 2023 among children aged 12-23 months selected from the urban and rural field-practice areas of Osmania Medical College, Hyderabad, Telangana, India, after obtaining approval from the Institutional Ethics Committee of Osmania Medical College (approval number: ECR/300/Inst/AP/2013/RR-16).

Study population and sample size

Children aged 12-23 months whose parents or caregivers provided written informed consent were included. Children from migrant families and those without an immunization card were excluded.

Sample size was calculated using the formula 4PQ/L², based on the full immunization coverage in Telangana (69.6%) reported by the National Family Health Survey-5 (NFHS-5) [12]. The minimum required sample size was 339; after accounting for a 10% non-response rate, this increased to 373 and was rounded to 400, with equal representation from urban (n=200) and rural (n=200) areas.

Sampling procedure

A multistage sampling technique was used. In the first stage, 10 of the 30 slums under the Urban Health Centre (UHC) and 10 of the 32 villages under the Rural Health Centre (RHC) were selected by simple random sampling using computer-generated random numbers. In the second stage, line lists of children aged 12-23 months were prepared for the selected slums and villages; eligible children were assigned unique identification numbers and selected by simple random sampling proportional to the number of children in each area, until the required sample size was reached.

Data collection

Data was collected through face-to-face interviews using a pretested, semi-structured questionnaire developed from the literature and the NFHS-5 women's questionnaire. The questionnaire was developed to collect information on the child's immunization status and relevant sociodemographic characteristics, including maternal and child age, maternal education, parity, religion, SES, place of residence, sex, and birth order, and immunization history, obtained from parents or caregivers. The data-collection instrument was reviewed and pretested before the main study to assess clarity, comprehensibility, and feasibility, and necessary modifications were made before final data collection.

For comparability with the NFHS vaccination indicator, a child aged 12-23 months was classified as fully immunized if they had received the eight basic vaccines comprising one dose of Bacille Calmette-Guérin (BCG), three doses of oral polio vaccine (OPV), three doses of DTP-containing vaccine, and one dose of measles-containing vaccine. This operational definition corresponds to the standard survey definition of full vaccination for children aged 12-23 months used in the NFHS/Demographic and Health Survey (DHS) schedule [13]. The definition was retained to ensure comparability with NFHS estimates and other population-based studies using the same indicator. This definition differs from the broader National Immunization Schedule (NIS) applicable during the study period, which included additional vaccines and doses. The study's estimate represents coverage according to the NFHS survey definition rather than complete adherence to all vaccines included in the contemporary NIS.

A fully immunized child was defined as one who had received one dose of BCG, three doses of OPV, three doses of combined DTP, and one dose of measles vaccine. Children were classified as fully immunized if they had received all vaccines specified in the study definition of full immunization. Children who had not completed this schedule were classified as non-fully immunized. Within the non-fully immunized group, children who had received no dose of a DTP-containing vaccine were identified as zero-dose, while those who had received at least one vaccine dose but had not completed the full schedule were considered partially immunized. The primary outcome was retained as a binary measure of full versus non-full immunization. Immunization status was verified using the Mother and Child Protection Card and cross-checked against records maintained at Anganwadi Centres to minimize recall bias.

Study variables

The primary outcome was full immunization status among children aged 12-23 months. Explanatory variables included sociodemographic, economic, and child-related characteristics (maternal age, maternal education, religion, SES, place of residence, and sex of the child).

Statistical analysis

Data was entered in Microsoft Excel (Microsoft Corp., Redmond, WA, USA) and analyzed in Stata version 19.0 (StataCorp LLC, College Station, TX, USA). Continuous variables were summarized as median and interquartile range (IQR) and categorical variables as frequencies and percentages. Differences between fully and non-fully immunized children were assessed using the chi-squared test for categorical variables and the Mann-Whitney U test for continuous variables. Statistical significance was set at p<0.05.

Socioeconomic inequality in full immunization coverage was assessed using the CI, a rank-dependent measure that quantifies the distribution of a health outcome across the socioeconomic class [14]. SES was assessed using the modified BG Prasad socioeconomic classification based on per-capita monthly household income, using the income thresholds applicable in 2023 [15]. For the descriptive and regression analyses, the five socioeconomic categories were consolidated into two groups: upper SES (upper and upper-middle classes) and lower SES (middle, lower-middle, and lower classes). For concentration-based inequality analysis, children were ranked according to their per-capita monthly household income from lowest to highest socioeconomic position, and the resulting socioeconomic rank was used to calculate the CI, EI, and Wagstaff Index (WI) [14,16-18].

The CI ranges from −1 to +1 in its conventional formulation, with a value of zero indicating socioeconomic equality. Positive values indicate that full immunization is concentrated among children with higher socioeconomic positions (pro-rich distribution), whereas negative values indicate concentration among children with lower socioeconomic positions (pro-poor distribution) [16]. However, because full immunization was measured as a binary, bounded outcome (0=not fully immunized; 1=fully immunized), the attainable bounds of the conventional CI depend on the mean level of the outcome and are not fixed at −1 and +1 [17]. Accordingly, the EI, a corrected CI designed for bounded health variables, was calculated [16]. The EI has a symmetric range from −1 to +1, with zero indicating equality and positive and negative values indicating pro-rich and pro-poor distributions, respectively.

The WI was also calculated as an alternative normalization of the conventional CI for a binary health outcome [18]. The WI was reported as a complementary measure to examine whether the direction and interpretation of socioeconomic inequality were consistent across alternative normalization approaches. The EI was specified as the primary corrected measure because it directly addresses the bounded nature of the binary immunization outcome, while the conventional CI and WI were reported as complementary measures. For all concentration-based measures, positive values indicated a pro-rich distribution of full immunization and negative values indicated a pro-poor distribution.

The absolute socioeconomic equity gap was calculated as the difference in full immunization coverage between children in the upper and lower socioeconomic groups. To quantify the contribution of measured explanatory factors to the observed socioeconomic gap in full immunization coverage, Fairlie decomposition was performed. Fairlie decomposition extends the Blinder-Oaxaca decomposition framework to binary outcomes using nonlinear logit or probit models [19]. The decomposition estimated the proportion of the observed socioeconomic gap explained by differences in the measured characteristics between the socioeconomic groups. Contributions were expressed as percentage contributions to the observed gap, with positive values indicating factors that widened the gap and negative values indicating factors that narrowed the gap. The model included the prespecified explanatory variables: maternal age, maternal literacy, birth order, religion, place of residence, and sex of the child. The decomposition results are reported as contributions to the explained component and are distinguished from the overall proportion of the observed socioeconomic gap explained by the model.

## Results

A total of 400 children were included, of whom 228 (57.0%) were fully immunized and 172 (43.0%) were not fully immunized. Full immunization was significantly associated with SES, place of residence, sex of the child, and birth order. Coverage was higher among children from the upper socioeconomic class (152/224, 67.86%) than among those from the lower socioeconomic class (76/176, 43.18%; p<0.001), among urban children (130/200, 65.0%) than rural children (98/200, 49.0%; p=0.002), and among males (140/226, 61.95%) than females (88/174, 50.57%; p=0.030). Coverage was lower among first-born children (124/236, 52.5%) than among children of higher birth order (104/164, 63.4%; p=0.030). Maternal age, maternal education, and religion were not significantly associated with immunization status (Table 1).

**Table 1:** Sociodemographic characteristics according to immunization status among children aged 12-23 months * indicates statistically significant at a p-value of <0.05. IQR: interquartile range

Characteristic	Fully immunized n (%) (n=228)	Non-fully immunized n (%) (n=172)	P-value
Maternal age, years, median (IQR)	24 (22-27)	24 (23-27)	0.321
Maternal age: ≤21 years	30 (13.2)	19 (11.0)	0.629
Maternal age: >21 years	198 (86.8)	153 (89.0)	
Maternal education: illiterate	10 (4.4)	7 (4.1)	1.000
Maternal education: literate	218 (95.6)	165 (95.9)	
Religion: Hindu	142 (62.3)	92 (53.5)	0.096
Religion: non-Hindu	86 (37.7)	80 (46.5)	
Socioeconomic status: upper class	152 (66.7)	72 (41.9)	<0.001*
Socioeconomic status: lower class	76 (33.3)	100 (58.1)	
Residence: urban	130 (57.0)	70 (40.7)	0.002*
Residence: rural	98 (43.0)	102 (59.3)	
Sex: male	140 (61.4)	86 (50.0)	0.030*
Sex: female	88 (38.6)	86 (50.0)	
Birth order: first	124 (62.0)	112 (56.0)	0.030*
Birth order: more than one	104 (52.0)	60 (30.0)	

The equity-coverage plane demonstrated substantial heterogeneity in socioeconomic equity across subgroups (Figure 1). Urban children had relatively high full immunization coverage (130/200, 65.0%) but the largest pro-rich socioeconomic gap (+52.7 percentage points), indicating that higher coverage coexisted with marked socioeconomic inequality. Females had lower overall coverage (88/174, 50.57%) but a relatively large pro-rich gap (+34.2 percentage points), while children of literate mothers had a gap of +26.5 percentage points. Males had higher coverage (140/226, 61.95%) with a smaller pro-rich gap (+18.6 percentage points). In contrast, rural children and children of illiterate mothers showed reversed socioeconomic gradients, with equity gaps of −5.1 and −11.5 percentage points, respectively. Overall, these findings indicate that higher immunization coverage does not necessarily correspond to greater socioeconomic equity, with urban children representing the most prominent subgroup with high coverage but substantial socioeconomic inequality.

**Figure 1:**
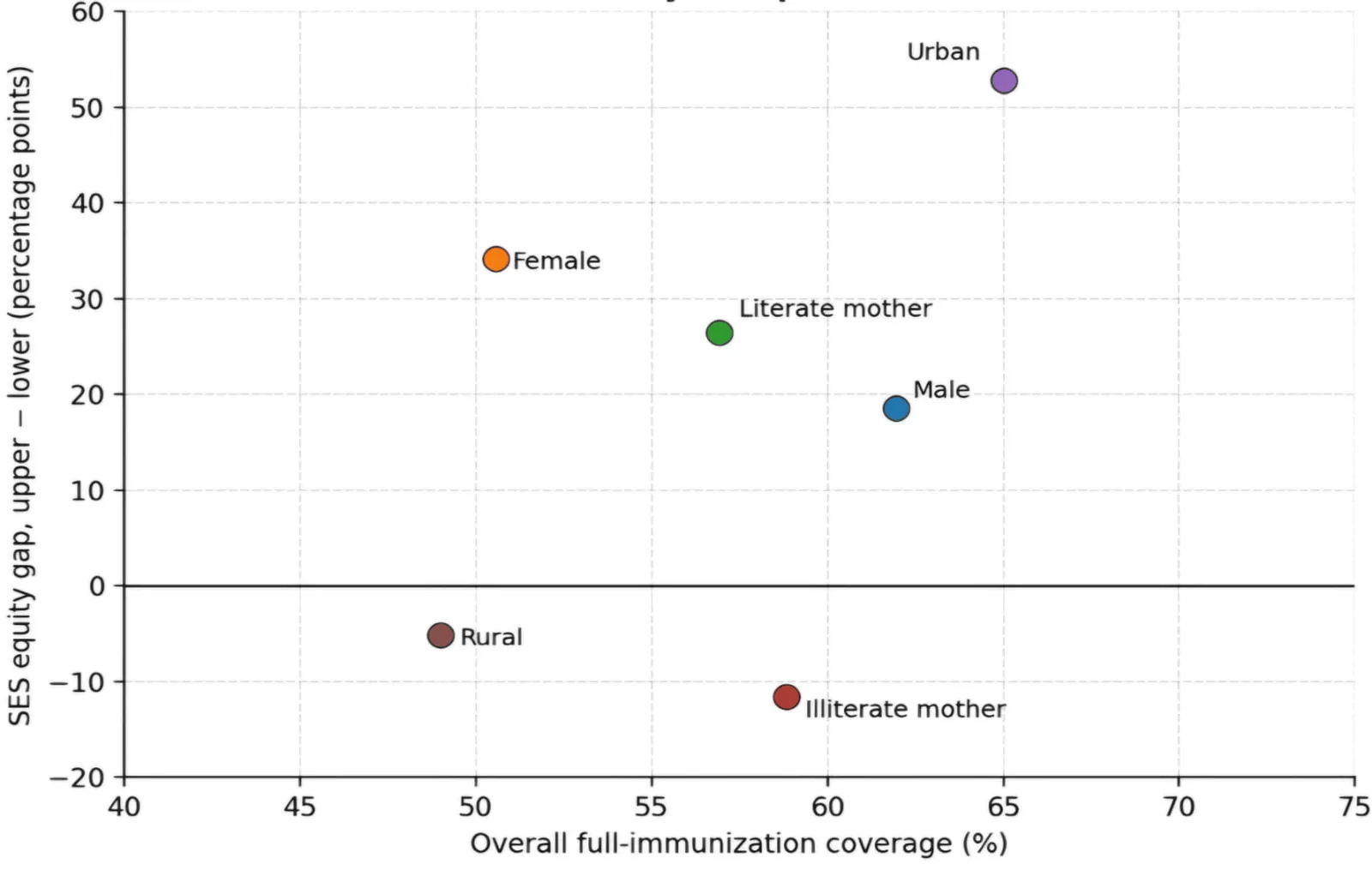
Equity-coverage plane illustrating socioeconomic inequality in full immunization by child sex, maternal literacy, and place of residence Points above the zero line indicate higher coverage among the upper socioeconomic group; points below the zero line indicate higher coverage among the lower socioeconomic group. SES: socioeconomic status

A socioeconomic gradient in full immunization was observed, with coverage being higher among children from the upper socioeconomic class than among those from the lower socioeconomic class (152/224 (67.86%) vs. 76/176 (43.18%)), corresponding to an absolute difference of 24.68 percentage points. The positive CI (0.1067) indicated a pro-rich distribution of full immunization, which was further supported by the positive EI (0.2432) and WI (0.2481) (Table 2).

**Table 2:** Socioeconomic inequality in full immunization coverage SES: socioeconomic status; CI: Concentration Index; EI: Erreygers Index; WI: Wagstaff Index

Indicator	Estimate
Total sample	400
Fully immunized	228/400 (57.0%)
Non-fully immunized	172/400 (43.0%)
Full immunization coverage: upper SES	152/224 (67.86%)
Full immunization coverage: lower SES	76/176 (43.18%)
Absolute socioeconomic gap (upper-lower)	24.68 percentage points
CI	+0.1067
EI	+0.2432
WI	+0.2481

Subgroup analysis demonstrated marked heterogeneity in socioeconomic disparities in full immunization coverage (Table 3). The overall equity gap was 24.7 percentage points, with higher coverage among children from the upper-SES group. The disparity was greatest among urban children (+52.7 percentage points) and females (+34.2 percentage points). A similar pro-rich gradient was observed among children of literate mothers and among Hindu and Muslim children. In contrast, a reversed socioeconomic gradient was observed among rural children, children of illiterate mothers, and Christian children, where coverage was higher in the lower-SES group. These findings indicate substantial heterogeneity in socioeconomic inequity in immunization coverage across population subgroups.

**Table 3:** Additional equity-coverage stratification by residence and religion SES: socioeconomic status

Subgroup	Overall coverage (%)	Upper SES (%)	Lower SES (%)	SES equity gap (pp)
Male	61.95	70.49	51.92	+18.57
Female	50.57	64.71	30.56	+34.15
Literate mother	56.92	68.18	41.72	+26.46
Illiterate mother	58.82	50.00	61.54	−11.54
Urban	65.00	83.72	30.99	+52.74
Rural	49.00	46.32	51.43	−5.11
Overall	57.0	67.9	43.2	+24.7
Religion: Hindu	60.7	71.7	42.7	+29.0
Religion: Muslim	54.9	74.5	35.3	+39.2
Religion: Christian	46.9	35.7	55.6	−19.8

Fairlie decomposition showed that urban residence made the largest contribution to widening the socioeconomic gap in full immunization coverage (136.9%), followed by Hindu religion (60.1%) and maternal age ≤21 years (6.3%). In contrast, first birth order (−53.1%), male sex (−25.4%), and maternal literacy (−24.8%) contributed in the direction of narrowing the gap (Table 4). Thus, 92.1% of the observed socioeconomic disparity was not explained by the measured covariates included in the model, suggesting that additional unmeasured socioeconomic and contextual factors may contribute to the observed disparity.

**Table 4:** Fairlie decomposition of the socioeconomic gap in full immunization

Determinant	Logistic coefficient	Standard error	Contribution to explained gap (%)	Direction
Maternal age ≤21 years	0.252	0.328	6.3	Widens
Maternal literacy	−0.389	0.522	−24.8	Narrows
First birth order	−0.423	0.216	−53.1	Narrows
Hindu religion	0.372	0.211	60.1	Widens
Urban residence	0.683	0.212	136.9	Widens
Male child	0.455	0.211	−25.4	Narrows
Total explained component	-	-	100.0	-

The observed socioeconomic gap in full immunization coverage was 24.68 percentage points, of which 1.96 percentage points (7.9%) was explained by the measured determinants included in the model (Table 5). Thus, 92.1% of the observed socioeconomic disparity was not explained by the measured covariates included in the model, suggesting that additional unmeasured socioeconomic and contextual factors may contribute to the observed disparity.

**Table 5:** Summary of socioeconomic inequality in full immunization coverage SES: socioeconomic status

Measure	Result
Full immunization: upper SES	152/224 (67.86%)
Full immunization: lower SES	76/176 (43.18%)
Observed SES gap	24.68 percentage points
Model-based explained gap	1.96 percentage points
Proportion of observed gap explained	7.9%

Hypothetical potential impact analysis

The observed difference in full immunization coverage between the upper- and lower-SES groups was 24.68 percentage points. Fairlie decomposition showed that the measured covariates collectively accounted for 1.96 percentage points of this difference, corresponding to 7.9% of the observed socioeconomic gap, while 92.1% remained unexplained by the variables included in the model. Within the explained component, urban residence and Hindu religion contributed to widening the observed gap, whereas first birth order, male sex, and maternal literacy contributed to narrowing it. These percentages represent relative contributions to the explained component and should not be interpreted as causal effects.

To illustrate the potential magnitude of the observed socioeconomic disparity, a hypothetical potential-impact analysis was performed. This analysis estimated the change in overall full immunization coverage under the assumption that all children in the study population achieved the coverage observed among children in the upper-SES group. Under this hypothetical scenario, overall coverage would increase from 228/400 (57.0%) to 67.86%, corresponding to an absolute potential coverage gain of 10.86 percentage points. This estimate represents the potential coverage difference under the specified hypothetical assumption and should not be interpreted as a causal attributable risk, predicted intervention effect, or evidence that eliminating socioeconomic inequality would necessarily produce this increase in coverage.

## Discussion

This community-based study found that only 57.0% of children aged 12-23 months in the urban and rural field-practice areas of Osmania Medical College were fully immunized, well below the global 90% target, with a pronounced socioeconomic gradient favoring upper-SES households. Full immunization was significantly associated with SES, place of residence, and sex of the child, whereas maternal age, maternal education, and religion showed no significant bivariate association.

SES

The strong association between SES and full immunization observed in this study is consistent with the wider literature on vaccine equity. Full immunization coverage was 152/224 (67.86%) among children from the upper socioeconomic class compared with 76/176 (43.18%) among those from the lower socioeconomic class, corresponding to an absolute SES gap of 24.68 percentage points. Patenaude et al. reported a pro-rich CI of 0.140 for full immunization in India and a national absolute equity gap of 37.1 percentage points between the most and least advantaged wealth quintiles, which was larger than the gap observed in our more localized sample [10]. Using a similar equity framework, Ali et al., in a systematic review and meta-analysis of 108 studies from low- and middle-income countries, estimated that children in the poorest wealth quintile were 27% less likely to be fully vaccinated than those in the richest quintile, concluding that wealth disparity dominates and may drive other forms of vaccination inequity [11]. By contrast, in the historically underserved districts of Munger (Bihar) and Darrang (Assam), Sharma et al. found the concentration curve for full immunization to lie above the line of equality, indicating a pro-poor distribution that they attributed to intensive targeting of marginalized communities under Mission Indradhanush [20]. Together, these comparisons suggest that although wealth-based gradients in immunization access are common, their direction and magnitude may be shaped by the intensity and reach of local outreach programmes and that the present study area may not yet have benefited from comparable targeted interventions.

Place of residence

Full immunization coverage was higher among urban children (130/200, 65.0%) than rural children (98/200, 49.0%), consistent with the historically described "urban advantage," often attributed to greater physical proximity to health facilities. This pattern is not universal. Sharma et al. reported higher coverage in rural areas than in urban slums in Bihar and Assam, commonly explaining this finding by the concentration of the urban poor in informal settlements, where social exclusion and high opportunity costs may offset the nominal accessibility advantage of urban residence [20]. Moreover, Allan et al. reported a similar finding, with children in urban areas having 26% lower odds of full immunization than children in rural areas [21]. In the present study, the Fairlie decomposition identified urban residence as the single largest contributor to widening the socioeconomic gap (136.9% of the observed gap contribution). This suggests that the benefits of urban infrastructure may have been captured disproportionately by upper-SES households, whereas lower-SES urban children may experience barriers characteristic of the "urban slum phenomenon" rather than simply barriers related to geographic access.

Gender of the child

A statistically significant sex difference in full immunization was observed, with 140/226 (61.95%) male children and 88/174 (50.57%) female children fully immunized (p=0.030). This direction is consistent with the pooled global estimate that girls are 3% less likely to be fully vaccinated than boys, although the magnitude of the difference in this study was considerably larger. This finding may indicate that gender-related differences in healthcare utilization are particularly relevant in this population. Mansilla et al. identified multiple barriers contributing to incomplete immunization, including systemic discrimination against women, limited political commitment to addressing gender disparities, inadequate sex-disaggregated and gender data, restrictive social norms, household responsibilities that limit women's autonomy and access to services, and misconceptions and stigma among families and peers [22]. Vidal Fuertes et al. found that the Gender Development Index was significantly associated with the proportion of zero-dose children, with high gender inequality associated with greater odds of zero-dose status (AOR=1.61) [23]. Notably, in the Fairlie decomposition, male sex narrowed rather than widened the socioeconomic gap (−25.4%), as did first birth order (−53.1%), implying that the observed sex- and birth-order-related patterns may have interacted with socioeconomic position. These findings should be interpreted cautiously because decomposition coefficients represent contributions to the socioeconomic gap rather than independent causal effects.

Maternal education and age

Neither maternal education nor maternal age was significantly associated with full immunization in this sample, which differs from substantial literature identifying maternal education as an important correlate of immunization inequality. Patenaude et al., for example, identified maternal education as a leading contributor to unfair disadvantage in national Indian data [10]. Geweniger et al. found that children whose mothers had secondary or higher education had approximately five times the odds of full vaccination compared with children whose mothers had no education (crude OR: 5.31; 95% CI: 2.77-10.17) [24]. In the present study, 218/383 (56.92%) children of literate mothers and 10/17 (58.82%) children of illiterate mothers were fully immunized. The most plausible explanation is a ceiling effect: more than 95% of mothers in both immunization-status groups were classified as literate, resulting in minimal variation with which to detect an association. This should be interpreted as a feature of the sampled population rather than evidence against the broader literature. It also highlights a limitation of dichotomizing maternal education into literate and illiterate categories rather than considering the level of schooling completed.

Religion

Religion was not significantly associated with immunization status in the bivariate analysis. Among fully immunized children, 142/234 (60.68%) were from Hindu households and 86/166 (51.81%) were from non-Hindu households; the corresponding comparison was not statistically significant (p=0.096). Syed et al. highlighted the potential role of faith-based organizations in public health campaigns and vaccination initiatives to support vaccine uptake and confidence [25]. However, in the Fairlie decomposition, Hindu religion was the second-largest contributor to widening the socioeconomic gap (60.1%). Thus, although religion alone was not a statistically significant predictor of immunization status, its contribution to the socioeconomic disparity suggests that it may interact with socioeconomic circumstances in ways that warrant further investigation, particularly given the modest subgroup sizes.

Measuring inequality: concentration and equity indices

The positive CI (+0.1067), EI (+0.2432), and WI (+0.2481) indicate a pro-rich distribution of full immunization in this population, in the same direction as, although smaller in magnitude than, the national estimate reported by Patenaude et al. [10]. The absolute socioeconomic gap was 24.68 percentage points, with coverage of 152/224 (67.86%) among upper-SES children compared with 76/176 (43.18%) among lower-SES children. The use of concentration and corrected indices alongside the absolute equity gap provides complementary measures of socioeconomic inequality in this binary health outcome.

Decomposing the gap: Fairlie decomposition

The Fairlie decomposition explained 1.96 percentage points (7.9%) of the observed 24.68-percentage-point socioeconomic gap, indicating that most of the disparity was not accounted for by the measured sociodemographic characteristics. Urban residence and Hindu religion widened the gap, whereas maternal literacy, first birth order, and male sex narrowed it. Urban residence contributed +136.9%, Hindu religion +60.1%, and maternal age ≤21 years +6.3% to the gap, whereas first birth order (−53.1%), male sex (−25.4%), and maternal literacy (−24.8%) contributed in the opposite direction. The small explained fraction, together with the relatively large contributions of selected variables, suggests that unmeasured factors may account for a substantial proportion of the observed inequality. Potential factors include health-system supply-side constraints, caregiver decision-making autonomy, distance to services, opportunity costs, and quality of care. These findings should therefore be interpreted as a decomposition of the observed socioeconomic gap rather than evidence of causal effects.

Intersectionality: compounding disadvantage

Stratified equity analysis demonstrated substantial heterogeneity in socioeconomic equity across population subgroups. The SES equity gap was larger among females (+34.15 percentage points) than males (+18.57 percentage points). Overall full immunization coverage was 88/174 (50.57%) among females and 140/226 (61.95%) among males. The SES gap was also substantially larger among urban children (+52.74 percentage points) than rural children (−5.11 percentage points), despite higher overall coverage among urban children (130/200, 65.0%) than rural children (98/200, 49.0%). Children of literate mothers had full immunization coverage of 218/383 (56.92%) and a pro-rich SES gap of +26.46 percentage points, whereas children of illiterate mothers had coverage of 10/17 (58.82%) and a reversed SES gradient of −11.54 percentage points. These findings demonstrate that socioeconomic inequality varies considerably across population subgroups and cannot be fully characterized by the overall SES gap alone. They also identify lower-SES girls and lower-SES urban households as potentially important priority groups for targeted interventions.

Hypothetical potential impact analysis

Overall full immunization coverage was 228/400 (57.0%). If the observed SES gap were eliminated under the assumptions of the population impact analysis, overall coverage could increase by 10.86 percentage points, from 57.0% to 67.86%, corresponding to a population attributable fraction of 16.0%. This suggests that socioeconomic inequality represents a potentially important component of the observed under-immunization burden in this population. Reporting population-level impact measures such as the population attributable fraction complements descriptive measures such as concentration indices and absolute equity gaps and may strengthen the policy relevance of the equity analysis.

Strengths

This study combines a conventional bivariate analysis with concentration and equity indices appropriate for a binary, bounded outcome, Fairlie decomposition, subgroup equity-coverage analysis, and population impact measures, a comprehensive and methodologically current approach to characterizing immunization inequity, consistent with recommended practice. Restricting the sample to a defined field-practice area, with equal urban and rural representation and immunization status verified against Mother and Child Protection Cards and Anganwadi records, reduces recall bias relative to studies relying solely on caregiver report.

Limitations

This study has several limitations. It is a cross-sectional design that allows assessment of associations and socioeconomic disparities but does not establish causality. The study was conducted in selected urban and rural field-practice areas of a single medical college, which may limit generalizability to other populations and settings.

The urban and rural participants were equally allocated (200 each) to facilitate comparison. This allocation may not reflect the underlying distribution of the source population. Therefore, the overall full immunization coverage of 57.0% should be interpreted as an estimate for the study sample and not as a population-weighted estimate for Hyderabad.

Migrant families and children without available immunization cards were excluded. These children may differ from the included population in socioeconomic circumstances or access to immunization services. Their exclusion may have introduced selection bias, although its direction and magnitude cannot be determined because information on these children was unavailable.

Full immunization was defined using specified doses of BCG, OPV, DTP, and measles-containing vaccine. This operational definition may differ from the complete immunization schedule applicable during the 2023 study period and could affect the estimated coverage and inequality measures. In addition, zero-dose and partially immunized children were combined because the number of zero-dose children was very small. Thus, vaccination initiation and completion could not be assessed separately.

SES was assessed using the modified BG Prasad classification based on per-capita monthly household income. Categories were consolidated into upper- and lower-SES groups for the primary categorical analyses. This categorization may have reduced information on the socioeconomic gradient and should be considered when interpreting the inequality measures.

Some subgroup analyses involved small numbers, particularly mothers classified as illiterate (n=17). Therefore, subgroup-specific estimates and apparent reversed or large socioeconomic gradients may be imprecise and should be interpreted cautiously.

The Fairlie decomposition is a model-based decomposition of an observed socioeconomic gap and does not establish causal effects. The 7.9% explained component and individual variable contributions should therefore not be interpreted causally. The remaining unexplained component may include unmeasured household, behavioral, contextual, access-related, or health-system factors. The 10.86-percentage-point population-impact estimate is also hypothetical and should not be interpreted as a predicted intervention effect or causal population attributable fraction.

## Conclusions

Full immunization coverage in the study population was 57.0% and showed a clear pro-rich socioeconomic gradient, with additional differences by place of residence, sex, and birth order. The observed socioeconomic gap was 24.68 percentage points, of which the measured sociodemographic factors accounted for 1.96 percentage points (7.9%) in the Fairlie decomposition. Subgroup analyses showed heterogeneity in socioeconomic disparities, particularly among females and urban children.

Socioeconomic inequalities in childhood immunization were not fully contributed by the measured sociodemographic characteristics. There are several unexplained disparities that can be reflected as unmeasured household, behavioral, contextual, access-related, or health-system factors, although these factors were not directly assessed and cannot be considered causal explanations. The findings highlight the need for further research incorporating health-system, access, and contextual factors to better understand the mechanisms underlying socioeconomic inequalities in childhood immunization.
